# Orchestrating Chemo-Ferroptosis via Nonthermal Magnetocatalysis: A Cascade Amplification Paradigm for Breast Cancer Therapy

**DOI:** 10.34133/bmr.0341

**Published:** 2026-03-17

**Authors:** Wenjie Wang, Yixiao Li, Xingyu Ma, Tianyu Chen, Dongyang Zhao, Jiawei Zhai, Xinbei Fang, Wenting Chen, Zhongling Ma, Xiaojun Zhang

**Affiliations:** ^1^ Northwest University Chang An Hospital, Northwest University, Xi’an, Shaanxi, 710069, China.; ^2^ School of Medicine, Northwest University, Xi’an, Shaanxi Province, 710069, China.; ^3^ Key Laboratory of Resource Biology and Biotechnology in Western China, Ministry of Education; Provincial Key Laboratory of Biotechnology, College of Life Sciences, Northwest University, Xi’an, Shaanxi Province, 710069, China.; ^4^ Northwest University Chang An Hospital, Faculty of Life Sciences and Medicine, Northwest University, Xi’an, Shaanxi, 710069, China.; ^5^ Department of Oncology, Chang An District Hospital, Xi’an, Shaanxi, 710118, China.

## Abstract

Conventional tumor microenvironment-responsive nanotherapies are limited by their passive dependence on endogenous triggers, often resulting in suboptimal therapeutic efficacy due to tumor heterogeneity. Furthermore, monotherapy frequently induces drug resistance, highlighting the need for synergistic strategies targeting multiple cell death pathways. Here, we propose and experimentally validate a cascade amplification paradigm designed to overcome these intrinsic limitations by integrating a tumor microenvironment-priming stage with a remotely triggered external amplification stage. This paradigm is embodied in an alternating magnetic field-responsive iron–porphyrin metal–organic framework nanoplatform, termed DFT. Under acidic conditions, the DFT nanoplatform is primed, exhibiting a >3.5-fold accelerated corelease of doxorubicin and catalytic iron ions. Subsequent exposure to a low-intensity alternating magnetic field (10 mT, 40 kHz) operating in a strictly nonthermal regime serves as the external accelerator, markedly amplifying the Fenton-like catalytic process. This intensified oxidative burst synergistically coactivates ferroptosis and doxorubicin-induced apoptosis—2 mechanistically distinct pathways that collectively counteract drug resistance. In vivo validation demonstrated a 74.6% tumor growth inhibition—nearly a 2-fold enhancement compared with the passive DFT group (40.7%). Notably, this efficacy is achieved while remaining well within established clinical safety limits, thereby addressing the safety–efficacy trade-off inherent to conventional magnetic hyperthermia. Overall, this study establishes a robust and versatile paradigm that harnesses external physical fields not for thermal ablation, but as programmable tools to remotely regulate catalytic biochemistry. This nonthermal, field-driven strategy offers a promising and safer route to combating chemotherapy resistance.

## Introduction

Breast cancer remains a major global health challenge, wherein intrinsic heterogeneity and adaptive resistance mechanisms continually undermine the efficacy of conventional treatments, highlighting the urgent need for innovative therapeutic paradigms [1,2]. Among emerging strategies, oxidative stress-based therapies—particularly chemodynamic therapy—have attracted considerable attention owing to their ability to exploit the tumor microenvironment (TME) [3–5]. However, the therapeutic success of these approaches depends not only on the capacity to generate reactive oxygen species (ROS) but also on overcoming the intrinsic limitations of catalytic kinetics [6,7]. In conventional Fenton-like reactions, the reaction rate is severely constrained by the low concentration of endogenous hydrogen peroxide (H₂O₂) and the heterogeneous acidity of the TME [8,9]. As a result, the ROS generated are typically insufficient to surpass the robust antioxidant defenses of tumor cells, such as elevated glutathione (GSH) levels, leading to suboptimal therapeutic efficacy [10–12]. Consequently, the central challenge in chemodynamic therapy has shifted from simple ROS production to achieving spatiotemporally controlled and cascade-amplified ROS bursts capable of breaching the tumor’s defensive threshold [5,13,14].

To address this kinetic limitation, we propose a novel therapeutic framework termed cascade amplification. This approach involves 2 sequentially orchestrated stages. In the first stage, the TME serves as an endogenous primer to activate the therapeutic nanoplatform and facilitate the in situ accumulation of catalytic substrates. The second stage introduces a remotely and precisely controllable external physical field that acts as an accelerator, dramatically enhancing the catalytic reaction rate upon reaching the primed state and thereby triggering an avalanche-like ROS burst [14–16]. Among various external stimuli, alternating magnetic field (AMF) offers exceptional potential for clinical translation owing to their deep tissue penetration (>10 cm) and noninvasive nature [17,18]. Importantly, this strategy represents a conceptual departure from traditional magnetic hyperthermia, which primarily relies on thermal ablation, toward the exploration of sophisticated nonthermal bioeffects [19]. Recent studies have demonstrated that low-intensity AMFs can modulate nanocatalytic kinetics through distinct nonthermal mechanisms, including field-enhanced mass transport and accelerated electron transfer. These effects enable AMF to serve as an ideal remote accelerator in our proposed cascade amplification paradigm [2,6].

Realizing this cascade amplification paradigm requires a nanocarrier with finely tuned structural and functional precision. Metal–organic frameworks (MOFs), particularly those based on iron–porphyrin, represent an exceptionally suitable platform for this purpose [20,21]. Their intrinsic iron-based metal nodes act as catalytic centers for Fenton-like reactions [13], while their acid-labile coordination bonds enable degradation in response to the acidic TME. This pH sensitivity fulfills the first-stage priming requirement by facilitating the synchronous release of catalytic iron ions and a coloaded chemotherapeutic agent such as doxorubicin (DOX) [22,23]. Moreover, the strategic integration of chemotherapy-induced apoptosis with ferroptosis—a regulated, iron-dependent cell-death process driven by lipid peroxidation—offers a powerful synergistic route to circumvent therapeutic resistance [24,25]. Unlike monotherapies that are vulnerable to single-pathway blockade (e.g., apoptosis evasion), this dual-pathway engagement functions as a mechanistic compensatory mechanism, ensuring cytotoxic efficacy even when one death modality is compromised. The iron–porphyrin MOF platform thus inherently combines 3 critical roles—chemotherapy carrier, ferroptosis inducer, and catalytic core—within a single, intelligently designed nanotherapeutic entity.

Despite the strong conceptual rationale for merging these elements, a significant gap persists in current research. Existing studies remain largely segregated and are constrained by 2 fundamental limitations. First, TME-responsive MOFs are passively governed by the variable biochemical milieu of tumors, which restricts therapeutic reliability and efficacy [13,26]. Second, although AMF-enhanced nanotherapies are rapidly emerging [6,19], most face a fundamental limitation of magnetic hyperthermia—achieving adequate therapeutic output often requires surpassing the Brezovich safety threshold (*H*·*f* ≤ 4.8 × 10^8^ A·m^−1^·s^−1^) [27]. Notably, even recent attempts to extend this limit to 1.9 × 10^9^ A·m^−1^·s^−1^ for brain tumor applications underscore the intrinsic conflict between safety and efficacy in thermally driven modalities [28]. These considerations highlight the pressing need for alternative, nonthermal strategies capable of balancing efficacy with safety. To date, no system has successfully integrated TME-responsive priming with strictly nonthermal, remotely controlled catalytic amplification within accepted clinical safety limits. Our approach directly addresses this unmet challenge by employing a low-intensity AMF (*H*·*f* = 3.18 × 10^8^ A·m^−1^·s^−1^)—well below the Brezovich limit—to achieve catalytic enhancement rather than thermal ablation. The key question, therefore, is how a low-intensity, nonthermal AMF can quantitatively enhance MOF-mediated Fenton reactions while preserving biocompatibility. This mechanism remains largely unexplored but holds significant promise for clinical translation.

**Fig. 1.**
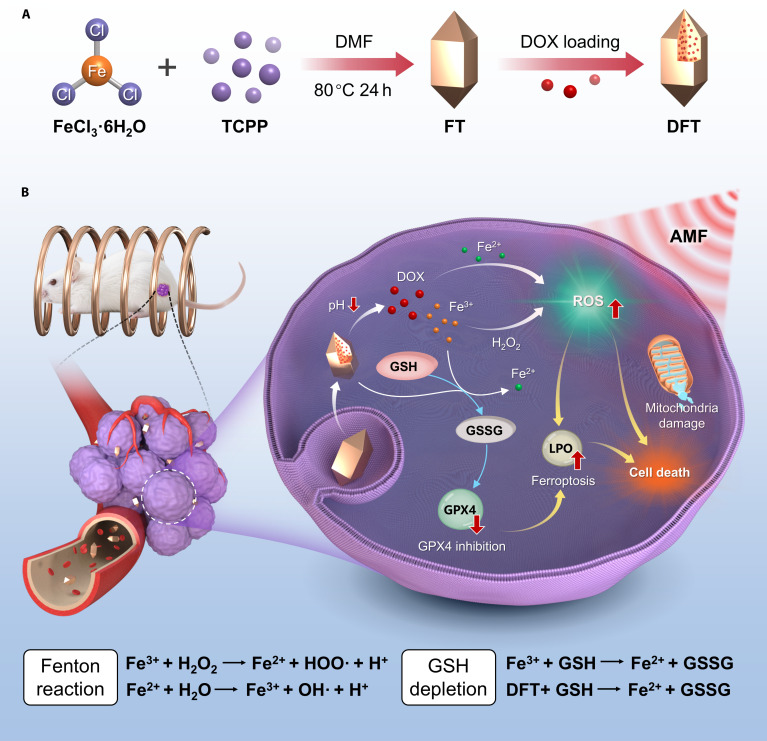
(A) Schematic illustration of the preparation of DFT nanoparticles. (B) Proposed mechanism of the DFT nanoplatform operating under the cascade amplification paradigm for synergistic breast cancer therapy, highlighting TME-responsive priming (corelease of DOX and Fe^3+^) and subsequent AMF-triggered amplification of intracellular ROS, leading to synergistic chemo-ferroptosis.

Herein, we bridge this knowledge gap by designing and validating an AMF-responsive iron–porphyrin MOF nanoplatform (DFT) (Fig. 1). This system was purpose-built to demonstrate the feasibility and therapeutic promise of the cascade amplification paradigm. In this system, the acidic TME functions as the endogenous primer to initiate corelease of DOX and catalytic Fe ions. Subsequently, exposure to a carefully optimized, low-intensity, nonthermal AMF acts as a remote accelerator, dramatically amplifying intracellular ROS generation. This oxidative burst is harnessed to robustly trigger ferroptosis while simultaneously enhancing susceptibility to DOX-induced apoptosis. Collectively, our results confirm the pivotal role of nonthermal AMF in enhancing synergistic antitumor efficacy. This finding establishes a foundational design principle for next-generation, remotely programmable nanomedicines with precise biochemical controllability.

## Materials and Methods

### Materials

Ferric chloride hexahydrate (FeCl₃·6H₂O), tetrakis(4-carboxyphenyl) porphyrin (TCPP), *N*,*N*-dimethylformamide (DMF), DOX hydrochloride, 3,3′,5,5′-tetramethylbenzidine (TMB), and 5,5′-dithiobis (2-nitrobenzoic acid) (DTNB) were purchased from Macklin Biochemical Technology (Shanghai, China). RMPI 1640 medium and fetal bovine serum (FBS) were obtained from HyClone (USA) and Cell-Box Biological Products Trading (Changsha, China), respectively. The Cell Counting Kit-8 (CCK-8) and penicillin–streptomycin were purchased from New Cell Molecular Biotech (Suzhou, China). The ROS detection kit, JC-1 mitochondrial membrane potential probe, 4′,6-diamidino-2-phenylindole, calcein acetoxymethyl ester (calcein-AM)/propidium iodide (PI) assay kit, and MitoTracker Green were obtained from Beyotime Biotechnology (Shanghai, China). The C11-BODIPY581/591 lipid peroxidation probe was purchased from Thermo Fisher Scientific (Shanghai, China). All other reagents were of analytical grade and used as received unless otherwise specified.

### Synthesis and characterization of nanoparticles

#### Synthesis of iron–porphyrin MOF and DFT nanoparticles

Iron–porphyrin MOF (FT) nanoparticles were synthesized through a solvothermal method adapted from a previously reported procedure [22]. Briefly, FeCl₃·6H₂O (20 mg) and TCPP (50 mg) were dissolved in 10 ml of DMF and stirred overnight at room temperature. Subsequently, an aqueous solution of FeCl₃·6H₂O was added to the TCPP solution, followed by the addition of 2 ml of glacial acetic acid (37 wt%). The mixture was stirred for another 2 h, after which 50 ml of DMF was introduced. The resulting solution was transferred to a Teflon-lined stainless steel autoclave and heated at 80 °C for 24 h in the dark. After cooling to room temperature, the FT product was collected by centrifugation (9,000 rpm, 20 min) and purified by sequential washing with DMF, methanol, and deionized water.

For drug loading, the purified FT nanoparticles (2 mg) were dispersed in 2 ml of ethanol using sonication. A solution of DOX (4 mg) in 2 ml of ethanol was then added to the FT dispersion, and the mixture was magnetically stirred in the dark at room temperature for 24 h to facilitate drug adsorption. The resulting DOX-loaded FT nanoparticles (denoted as DFT) were collected by centrifugation and washed 3 times with ethanol to remove nonencapsulated DOX.

#### Physicochemical characterization

The morphology and nanostructure of the FT and DFT nanoparticles were characterized using scanning electron microscopy (SEM; JSM-7610F Plus, JEOL) and transmission electron microscopy (TEM; JEM-2100, JEOL). The hydrodynamic size distribution and zeta potential were determined by dynamic light scattering (DLS; Zetasizer Nano ZS90, Malvern Instruments). The crystalline structure was analyzed by powder x-ray diffraction (PXRD; SmartLab SE, Rigaku). The chemical structure and elemental composition were evaluated by Fourier-transform infrared spectroscopy (FTIR; TENSOR 27, Bruker) and x-ray photoelectron spectroscopy (XPS; ESCALAB 250Xi, Thermo Fisher Scientific). The optical absorption characteristics were examined using ultraviolet–visible (UV–vis) spectroscopy (UV-2500, Shimadzu).

#### Drug loading content and encapsulation efficiency

The amount of DOX loaded onto the FT nanoparticles was quantified by measuring the absorbance of unencapsulated DOX in the supernatants collected during the washing process using UV–vis spectroscopy at 480 nm. The encapsulation efficiency and drug loading content were calculated according to the following equations:

$$\begin{array}{c} \begin{array}{c} \text{Encapsulation efficiency}\;\left(\%\right)=\\ {}[(\text{Weight of initial}\;\mathrm{DOX}-\text{Weight of}\;\mathrm{DOX}\;\text{in supernatant})/\\ \text{Weight of initial}\;\mathrm{DOX}]\times 100\% \end{array} \end{array}$$ (1)

$$\begin{array}{c} \begin{array}{c} \begin{array}{c} \text{Drug loading content}\;\left(\%\right)=\\ {}[(\text{Weight of initial}\;\mathrm{DOX}-\text{Weight of}\;\mathrm{DOX}\;\text{in supernatant})/\\ \text{Total weight of}\;\mathrm{DFT}\;\text{nanoparticles}]\times 100\% \end{array} \end{array} \end{array}$$ (2)

### In vitro functional assays for TME responsiveness

#### In vitro release of DOX and iron ions

The in vitro release profiles of DOX and iron ions from DFT nanoparticles were investigated in phosphate-buffered saline (PBS) at pH 7.4 (simulating physiological conditions) and pH 5.2 (simulating the acidic TME). Briefly, DFT nanoparticles (1 mg) were dispersed in 20 ml of the respective PBS solution and incubated at 37 °C with gentle shaking. At predetermined time intervals, 2-ml aliquots of the release medium were collected and replaced with an equal volume of fresh buffer to maintain sink conditions. The concentration of released DOX was quantified by UV–vis spectroscopy at 480 nm, while the concentration of iron ions was determined using inductively coupled plasma mass spectrometry.

#### Assessment of peroxidase-like catalytic activity

The pH-dependent generation of hydroxyl radicals (•OH) was evaluated using TMB as a chromogenic substrate. In a typical assay, DFT nanoparticles (final concentration: 10 mg/ml) were incubated with TMB (0.2 mM) and H₂O₂ (2 mM) in buffer solutions of either pH 5.2 or pH 7.4. The reactions were carried out at 37 °C in the dark. The oxidation of TMB induced by •OH radicals was monitored by measuring the absorbance of the reaction solution at 652 nm at predetermined time points using a microplate reader.

#### GSH consumption assay

The pH-dependent GSH-depleting capability of DFT nanoparticles was examined using DTNB as an indicator reagent. DFT nanoparticles were suspended in buffer solutions (pH 5.2 or 7.4) containing 10 mM GSH and incubated at 37 °C under constant shaking. At designated time intervals, aliquots were collected and centrifuged to remove the nanoparticles. The supernatant was subsequently mixed with a DTNB solution (10 mg/ml) and incubated for 5 min. The reduction in GSH concentration was quantified by measuring the decrease in absorbance of the yellow product, 2-nitro-5-thiobenzoate, at 412 nm using a microplate reader.

### In vitro cellular assays

#### Cell culture

Murine breast cancer (4T1) cells and L929 murine fibroblasts were cultured in RPMI 1640 medium supplemented with 10% FBS and 1% penicillin–streptomycin. All cells were maintained at 37 °C in a humidified incubator containing 5% CO₂.

#### Cellular internalization

The cellular uptake of DFT nanoparticles by 4T1 cells was examined using confocal laser scanning microscopy (CLSM). Cells (2 × 10^5^ per dish) were seeded onto confocal culture dishes, incubated overnight, and then exposed to DFT nanoparticles (40 μg/ml) for 2 or 4 h. Following incubation, the cells were fixed with 4% paraformaldehyde, and the nuclei were counterstained with 4′,6-diamidino-2-phenylindole. Fluorescence images were acquired using a Leica TCS SP8 CLSM.

#### Evaluation of AMF-potentiated synergistic cytotoxicity

To evaluate synergistic cytotoxicity, 4T1 cells were treated with one of the following formulations: control, FT, DFT, AMF alone, or AMF + DFT. The final concentration of DFT was 40 μg/ml. For AMF-treated groups, a low-intensity AMF (10 mT, 40 kHz, 40 min)—optimized in preliminary experiments (see the Supplementary Materials)—was applied immediately after nanoparticle administration.

##### Cell viability assay

Cell viability was quantified using the CCK-8 assay. Cells were seeded in 96-well plates (10,000 cells per well), treated for 24 h as described above, and then incubated with CCK-8 solution for 2 h. Absorbance was measured at 450 nm using a microplate reader.

##### Live/dead staining

Cell membrane integrity and viability were visualized using a calcein-AM/PI assay kit. After 12 h of treatment, cells cultured on glass-bottom dishes were costained with calcein-AM (live, green) and PI (dead, red), followed by imaging with CLSM.

##### Clonogenic assay

The long-term proliferative potential of treated 4T1 cells was assessed by colony formation. Cells (1,000 cells per well) were seeded in 24-well plates, exposed to the designated treatments, and cultured in fresh medium for 7 d. The resulting colonies were fixed and stained with 0.1% crystal violet for visualization and quantification.

#### Mechanistic assays for apoptosis and ferroptosis

##### Intracellular ROS production

Intracellular ROS levels were assessed using the 2′,7′-dichlorodihydrofluorescein diacetate (DCFH-DA) probe. After 12 h of treatment, 4T1 cells were incubated with DCFH-DA (10 μM) for 30 min at 37 °C. The cells were then washed with PBS, and the fluorescence of oxidized DCF was observed by CLSM (excitation: 488 nm; emission: 516 nm).

##### Mitochondrial membrane potential analysis

Changes in mitochondrial membrane potential (MMP) were evaluated using the JC-1 fluorescent probe. Following 12 h of treatment, cells were incubated with JC-1 (2 μM) for 30 min at 37 °C. The red fluorescence (JC-1 aggregates, indicating high MMP) and green fluorescence (JC-1 monomers, indicating MMP depolarization) were visualized by CLSM. The ratio of green-to-red fluorescence intensity was calculated to quantify mitochondrial depolarization.

##### Lipid peroxidation assays

Lipid peroxidation was analyzed using both qualitative and quantitative approaches. For fluorescence imaging, cells were treated for 12 h, stained with the lipid peroxidation sensor C11-BODIPY581/591, and imaged by CLSM. For quantitative evaluation, the intracellular level of malondialdehyde (MDA)—a terminal product of lipid peroxidation—was determined using a commercial MDA assay kit, following the manufacturer’s instructions.

##### Western blot analysis

The expression levels of apoptosis- and ferroptosis-related proteins, including glutathione peroxidase 4 (GPX4), Bax, and Bcl-2, were quantified by Western blot. Following 12 h of treatment, cells were lysed, and total protein concentrations were measured using a bicinchoninic acid assay. Equal protein amounts were separated by sodium dodecyl sulfate-polyacrylamide gel electrophoresis, transferred to polyvinylidene difluoride membranes, and probed with specific primary antibodies followed by horseradish peroxidase-conjugated secondary antibodies. Protein bands were visualized using an enhanced chemiluminescence detection kit, and relative band intensities were quantified with ImageJ software, using β-actin as the internal loading control.

### In vivo antitumor efficacy and biodistribution studies

#### Animal model and ethical statement

All animal experiments were performed in accordance with institutional guidelines for the care and use of laboratory animals and were approved by the Experimental Animal Ethics Committee of Northwest University (Approval No. ACUC2021045). Female BALB/c mice (6 to 8 weeks old, ~20 g) were obtained from the Experimental Animal Center of Air Force Medical University and housed under specific pathogen-free conditions. To establish the 4T1 tumor xenograft model, 1 × 10^6^ 4T1 cells suspended in 100 μl of PBS were subcutaneously injected into the right dorsal flank of each mouse. Experiments were initiated when tumor volumes reached approximately 100 mm^3^.

#### In vivo biodistribution study

To prepare the fluorescence-tracking probe, indocyanine green (ICG, 1 mg) was loaded onto DFT nanoparticles (2 mg) by stirring in 2 ml of deionized water for 8 h in the dark, followed by centrifugation and washing to remove unbound ICG. The resulting formulation was denoted as DFT@ICG. Tumor-bearing mice (*n* = 3 per group) were intratumorally injected with DFT@ICG at an ICG-equivalent dose of 5 mg/kg. For the AMF-enhanced targeting group, a localized low-intensity AMF (10 mT, 40 kHz, 40 min) was applied to the tumor region immediately after injection.

Whole-body near-infrared fluorescence imaging was conducted at predetermined intervals (preinjection, 4, 8, 12, 24, 36, 48, 72, and 96 h) using an IVIS Spectrum imaging system. Quantitative analysis of tumor accumulation was performed by calculating the mean fluorescence intensities of tumor sites and contralateral background regions using ImageJ, maintaining identical region-of-interest sizes. The tumor-to-background (T/B) ratio was determined by dividing these 2 values. At 24, 48, and 96 h postinjection, mice were euthanized, and major organs (heart, liver, spleen, lungs, and kidneys) and tumors were excised for ex vivo fluorescence imaging to confirm ICG biodistribution.

#### In vivo antitumor efficacy study

When tumor volumes reached approximately 100 mm^3^, mice were randomly divided into 5 treatment groups (*n* = 8 per group): (a) Saline (Control), (b) AMF only, (c) FT, (d) DFT, and (e) AMF + DFT. Treatments were administered via intratumoral injection twice weekly for 2 weeks. Doses were as follows: FT (an Fe-equivalent dose matching DFT), DFT, and AMF + DFT (5 mg/kg DOX equivalent). For AMF-treated groups, the tumor region was exposed to the optimized low-intensity AMF (10 mT, 40 kHz, 40 min) at 24 h postinjection to align with the time point of maximal tumor accumulation, ensuring optimal synchronization between payload bioavailability and magnetic field application.

Tumor volumes were calculated using the formula *V* = 0.5 × length × width^2^, and both tumor sizes and body weights were recorded every 2 d. At the end of the treatment period, mice were euthanized, and the tumors were excised, weighed, and photographed for quantitative evaluation.

#### Histological and immunohistochemical analysis

At the conclusion of the efficacy study, excised tumor tissues were fixed in 4% paraformaldehyde, embedded in paraffin, and sectioned into 5-μm slices. Tumor sections were stained with hematoxylin and eosin (HE) for morphological assessment. For mechanistic validation, immunohistochemical analysis was conducted using primary antibodies against Ki-67 (cell proliferation), terminal deoxynucleotidyl transferase dUTP nick end labeling (TUNEL) (apoptosis), and GPX4 (ferroptosis regulation).

### Biocompatibility and biosafety assessment

#### In vitro biocompatibility and hemocompatibility

The cytocompatibility of FT and DFT nanoparticles toward normal cells was evaluated using L929 murine fibroblasts and the CCK-8 assay. Cells were incubated with nanoparticles at gradient concentrations for 24 h, after which cell viability was determined according to the manufacturer’s protocol.

For the hemocompatibility evaluation, fresh red blood cells isolated from healthy BALB/c mice were incubated with DFT nanoparticles (5 to 80 μg/ml) at 37 °C for 4 h. Deionized water and PBS served as positive and negative controls, respectively. After incubation, samples were centrifuged, and the absorbance of released hemoglobin in the supernatant was measured at 570 nm to quantify hemolysis.

#### In vivo systemic biosafety assessment

Systemic biosafety was assessed at the completion of the in vivo therapeutic study. Whole blood samples were collected from each group for serum biochemistry analysis using an automatic biochemical analyzer to determine liver function markers—alanine aminotransferase (ALT), aspartate aminotransferase (AST), and alkaline phosphatase (ALP)—and kidney function markers—blood urea nitrogen (BUN) and creatinine (CREA). Concurrently, major organs (heart, liver, spleen, lungs, and kidneys) from all treatment groups were harvested, fixed in 4% paraformaldehyde, embedded in paraffin, sectioned, and stained with HE to assess potential histopathological abnormalities.

### Statistical analysis

All quantitative results are presented as means ± SD from at least 3 independent experiments, unless otherwise noted. Statistical analyses were performed using 1-way or 2-way analysis of variance followed by Tukey’s post hoc test for multiple comparisons (GraphPad Prism). A *P* value < 0.05 was considered statistically significant. Statistical significance levels in figures are indicated as follows: *P < 0.05, **P < 0.01.

## Results

### Synthesis and physicochemical characterization of the MOF-based nanoplatform

**Fig. 2.**
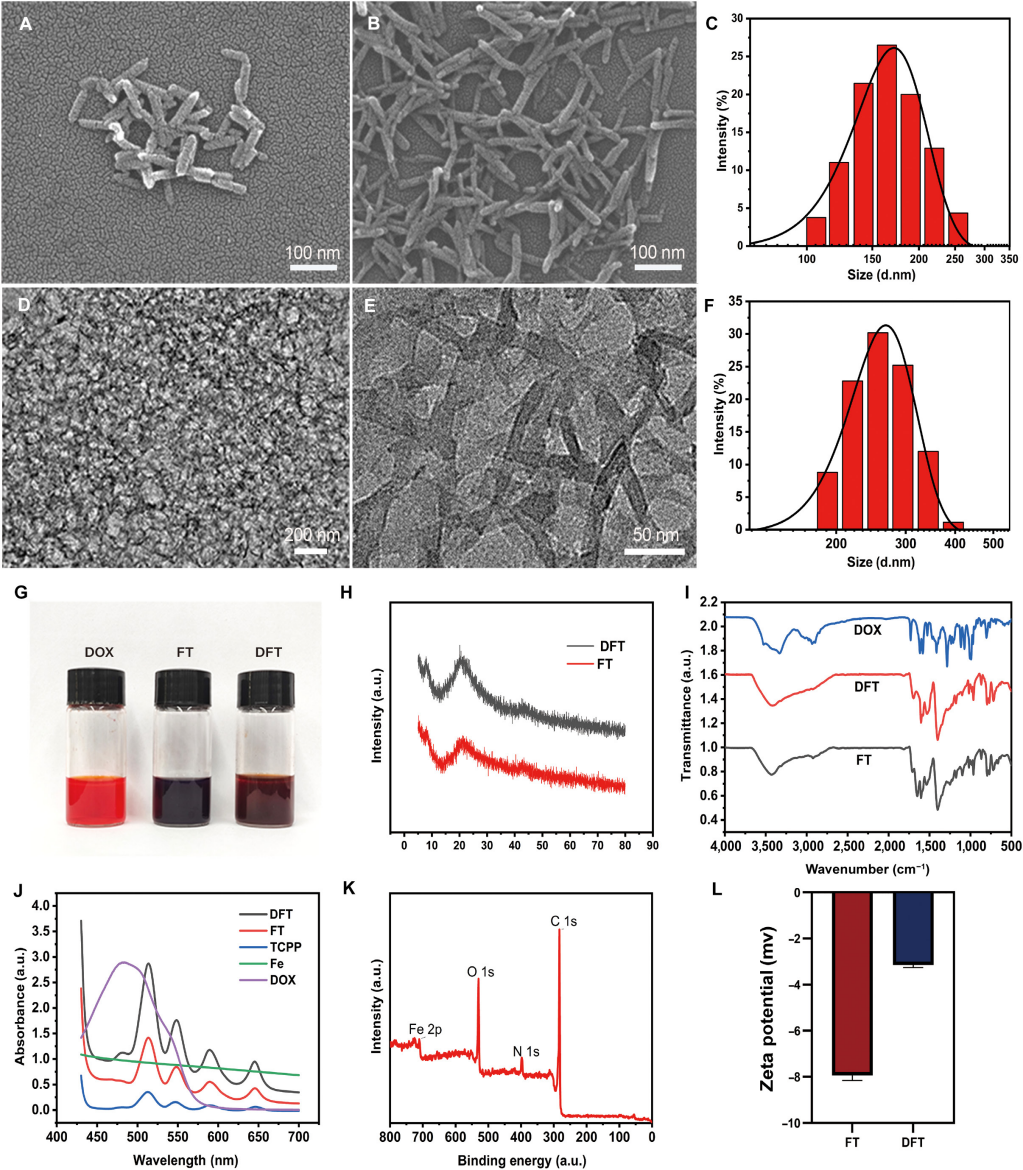
Physicochemical characterization of FT and DFT nanoparticles. (A) SEM image of FT. (B) SEM image of DFT. (C) Hydrodynamic size distribution of FT determined by DLS. (D and E) TEM images of DFT at different magnifications. (F) Hydrodynamic size distribution of DFT determined by DLS. (G) Photographs showing the color change from FT to DFT during DOX loading. (H) PXRD patterns of FT and DFT. (I) FTIR spectra of DOX, FT, and DFT. (J) UV–vis absorption spectra of TCPP, Fe^3+^, DOX, FT, and DFT. (K) XPS survey spectrum of DFT. (L) Zeta potentials of FT and DFT in ultrapure water.

To construct a nanoplatform capable of orchestrating the proposed cascade amplification, we synthesized FT via a solvothermal method. TEM/SEM revealed uniform, rod-like nanoparticles approximately 100 nm in length (Fig. 2A). DLS analysis showed a hydrodynamic diameter of ~164 nm (Fig. 2C), a size conducive to passive tumor accumulation through the enhanced permeability and retention effect while minimizing premature renal clearance. The chemotherapeutic agent DOX was subsequently loaded onto the FT framework through physical adsorption to yield the final DFT nanoplatform.

Successful DOX encapsulation was confirmed by comprehensive physicochemical analyses. Morphological observation by SEM and TEM demonstrated that DFT retained the uniform rod-like geometry of the parent FT nanoparticles (Fig. 2B, D, and E versus Fig. 2A). In aqueous dispersion, the hydrodynamic diameter increased from ~164 nm (FT; Fig. 2C) to ~246 nm (DFT; Fig. 2F), accompanied by a visible color transition of the suspension from brown to reddish-brown (Fig. 2G). Concurrently, the zeta potential shifted from −18.3 mV (FT) to −25.8 mV (DFT) (Fig. 2L), collectively indicating successful drug loading and surface modification.

The structural integrity of the MOF framework and the incorporation of DOX were further validated through crystallographic and spectroscopic analyses. PXRD patterns of DFT closely matched those of FT, confirming that the crystalline framework remained intact after drug loading—a prerequisite for controlled and predictable release (Fig. 2H). FTIR spectra of DFT displayed characteristic DOX peaks absent in the FT profile (Fig. 2I), while UV–vis spectra exhibited combined absorption features from both FT and DOX (Fig. 2J). XPS survey scans further confirmed the expected elemental composition of the final DFT nanoplatform (Fig. 2K).

The DOX loading content and encapsulation efficiency were determined to be 17.6% (w/w) and 35.2%, respectively. Moreover, 3 independently synthesized batches of DFT nanoparticles exhibited nearly identical physicochemical characteristics, including hydrodynamic diameters of 246 ± 8.5 nm and zeta potentials of −25.8 ± 1.2 mV, demonstrating excellent batch-to-batch reproducibility. Together, these findings confirm the successful synthesis of a structurally stable, well-defined DFT nanoplatform, providing a robust foundation for subsequent evaluations of its TME-responsive and therapeutic properties.

### The DFT nanoplatform is primed by the TME

#### pH-responsive corelease of DOX and iron ions

**Fig. 3.**
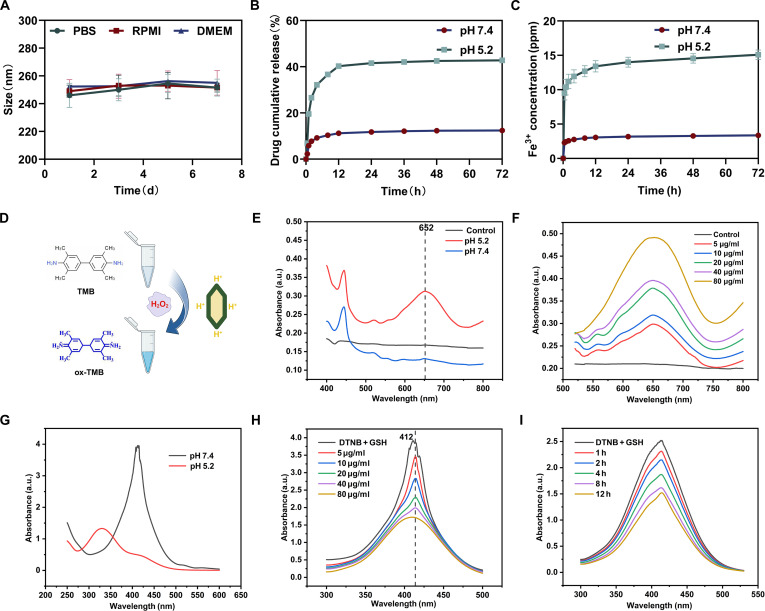
TME-responsive priming behavior of the DFT nanoplatform. (A) Colloidal stability of DFT over 7 d. (B) In vitro DOX release profiles from DFT at pH 7.4 and pH 5.2. (C) In vitro Fe^3+^ release profiles from DFT at pH 7.4 and pH 5.2. (D) Schematic illustration of •OH generation through a Fenton-like reaction. (E) pH-dependent •OH generation. (F) Concentration-dependent •OH generation. (G) pH-dependent GSH consumption. (H) Concentration-dependent GSH consumption. (I) Time-dependent GSH consumption. DMEM, Dulbecco’s modified Eagle’s medium; a.u., arbitrary units.

Following the successful construction of the DFT nanoplatform, its responsiveness to tumor-associated biochemical cues was systematically evaluated. The nanoplatform was engineered to undergo structural destabilization under acidic conditions characteristic of the TME. DFT demonstrated excellent colloidal stability in various physiologically relevant media, including PBS and cell-culture medium supplemented with 10% FBS, exhibiting negligible aggregation over 7 d (Fig. 3A). Such stability is crucial for maintaining prolonged systemic circulation and efficient tumor accumulation.

The pH-dependent release of the therapeutic payload was then investigated under conditions mimicking physiological (pH 7.4) and acidic (pH 5.2) environments. At pH 5.2, DFT released approximately 44.15% of its DOX payload within 72 h—a 3.5-fold increase over the ~12.37% released at pH 7.4 (Fig. 3B). This accelerated release at low pH is attributed to the acid-labile nature of the metal–ligand coordination bonds within the MOF framework. In parallel, Fe^3+^ ion release was substantially enhanced at pH 5.2, showing a 4.5-fold higher concentration than that measured at pH 7.4 after 72 h (Fig. 3C).

This synchronized, pH-triggered corelease of both the chemotherapeutic agent (DOX) and the catalytic species (Fe^3+^) in acidic conditions represents the foundational priming event of the proposed therapeutic cascade, ensuring preferential activation within the pathological TME while minimizing off-target effects in normal physiological environments.

#### pH-dependent pro-oxidative and GSH-depleting activity

The functional consequence of TME-triggered Fe^3+^ release is the initiation of pro-oxidative catalytic activity. To evaluate this property, the peroxidase-like capability of DFT to generate •OH through Fenton-like reactions was assessed using the TMB assay (Fig. 3D to F). In the presence of H₂O₂, DFT exhibited pronounced pH-dependent catalytic activity, producing robust TMB oxidation at pH 5.2 while showing negligible activity at physiological pH 7.4. These findings confirm that DFT possesses a potent, pH-switchable ability to catalytically decompose H₂O₂ into highly reactive •OH radicals—the foundational step of the TME-specific Fenton-like reaction.

Given that the effectiveness of ROS-based therapies is often mitigated by intracellular antioxidants, we next evaluated the ability of DFT to deplete reduced GSH, a major cellular redox regulator. DFT induced efficient, time- and concentration-dependent GSH consumption under acidic conditions (Fig. 3G to I). Within 60 min, treatment with 160 μg/ml DFT depleted approximately 83.4% of available GSH at pH 5.2, compared with only 31.2% at pH 7.4. This pH-selective GSH depletion serves a dual purpose: (a) It lowers the cellular antioxidant capacity, thereby reducing the threshold for oxidative damage; and (b) it sensitizes tumor cells to ferroptosis induction.

These results demonstrate that the DFT nanoplatform embodies all key functionalities required for the priming stage of the cascade: TME-selective corelease of therapeutic payloads, pH-gated generation of oxidizing •OH species, and suppression of intracellular redox defenses. These attributes establish the biochemical conditions necessary for subsequent therapeutic amplification upon external activation.

### AMF serves as a remote accelerator to amplify ROS generation and synergistic cytotoxicity

**Fig. 4.**
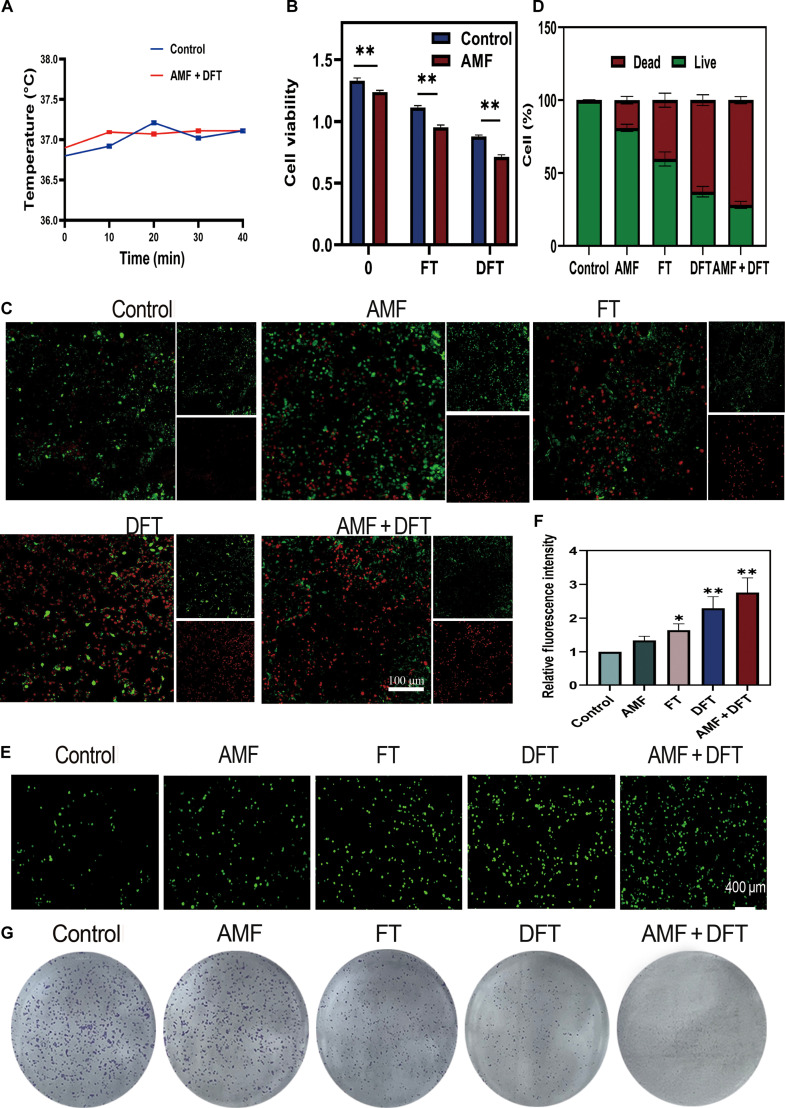
AMF acts as a remote accelerator to amplify DFT efficacy via ROS-mediated synergy. (A) Temperature profiles of cell-culture media containing DFT nanoparticles (40 μg/ml) recorded over 40 min with or without AMF exposure (10 mT, 40 kHz). (B) Cell viability of 4T1 cells following the indicated treatments. (C) Representative live/dead fluorescence images of 4T1 cells. Scale bar: 250 μm. (D) Quantitative analysis of the live/dead cell ratio. (E) CLSM images of intracellular ROS generation detected with DCFH-DA. Scale bar: 400 μm. (F) Quantification of ROS levels by relative fluorescence intensity. (G) Representative clonogenic assay images showing inhibition of long-term proliferation. All experiments were conducted under optimized conditions (DFT: 40 μg/ml; AMF: 10 mT, 40 kHz, 40 min). Data are presented as means ± SD. *P < 0.05, **P < 0.01.

Having established that the DFT nanoplatform can be effectively primed by the acidic TME, we next investigated whether AMF could function as a remote accelerator within the proposed cascade. To this end, 4T1 breast cancer cells were treated with DFT nanoparticles under optimized AMF conditions (10 mT, 40 kHz, 40 min), parameters that were systematically determined to maximize therapeutic synergy (Fig. S1). Importantly, in situ thermometric monitoring during AMF exposure showed a stable temperature–time profile, with no detectable increase above the physiological baseline. This confirms that the observed therapeutic enhancement occurred under a strictly nonthermal regime (Fig. 4A).

The synergistic cytotoxicity resulting from this dual-mode treatment was first evaluated using the CCK-8 assay. The combined AMF + DFT treatment reduced 4T1 cell viability to 48.3%, a significantly greater reduction than that observed for DFT alone (33.9%) or AMF alone (8.2%) (Fig. 4B). Notably, the observed cytotoxicity exceeded the predicted additive effect of the individual treatments, confirming a genuine synergistic interaction rather than a simple summation of effects. This finding was further corroborated by live/dead fluorescence staining, which revealed a markedly higher proportion of propidium-iodide-positive (dead) cells in the AMF + DFT group compared with all other controls (Fig. 4C and D).

To elucidate the mechanism underlying the observed synergistic cytotoxicity, we quantified intracellular ROS levels using the fluorescent probe DCFH-DA. While DFT or AMF treatment alone induced modest increases in ROS (2.2-fold and 1.3-fold relative to control, respectively), their combination (AMF + DFT) produced a striking 2.8-fold amplification of the intracellular ROS signal (Fig. 4E and F). This pronounced oxidative surge directly correlated with the enhanced cytotoxicity observed in the viability assays.

The long-term impact of this amplified cell killing was further assessed by clonogenic analysis, which revealed that the AMF + DFT treatment most effectively suppressed the proliferative potential of 4T1 cells (Fig. 4G). Collectively, these results provide direct evidence for the second, critical stage of the proposed cascade amplification paradigm.

The application of a low-intensity, nonthermal AMF serves as a potent remote activator, dramatically amplifying the ROS-generating capability of the TME-primed DFT nanoplatform. This externally triggered oxidative burst acts as the primary driver of the observed synergistic cytotoxicity.

### The amplified oxidative burst triggers synergistic apoptosis and ferroptosis

**Fig. 5.**
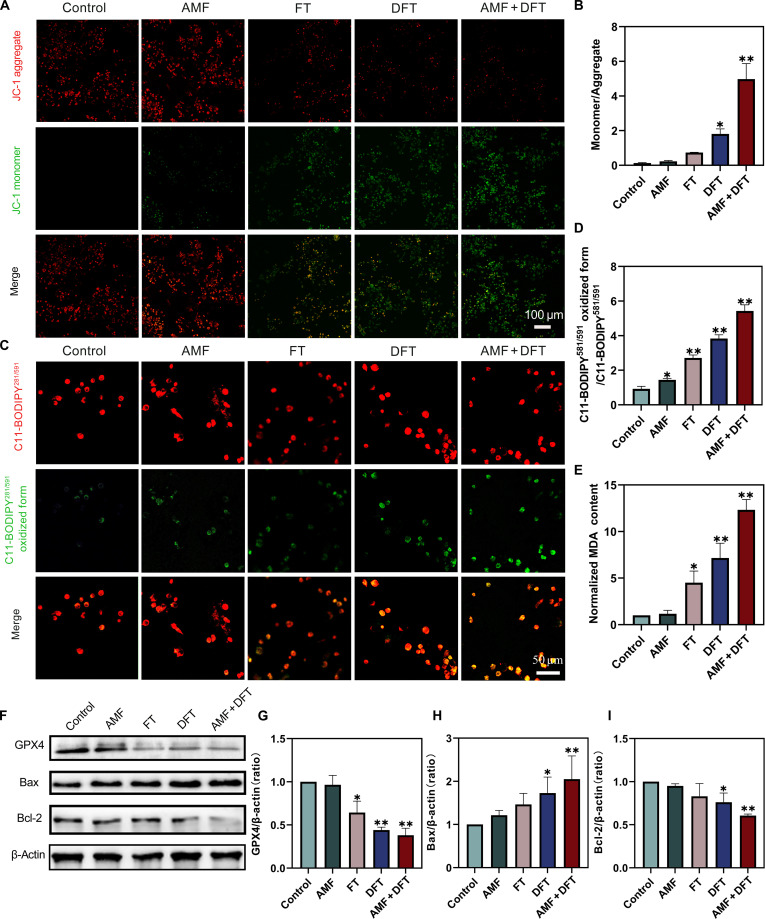
Mechanistic investigation of synergistic cell death: coactivation of apoptosis and ferroptosis. (A) CLSM images of MMP in 4T1 cells determined by JC-1 staining. Scale bar: 100 μm. (B) Quantitative analysis of MMP depolarization. (C) CLSM images of lipid peroxidation detected using C11-BODIPY^581^/^591^. Scale bar: 50 μm. (D) Quantification of intracellular lipid peroxidation levels. (E) Intracellular MDA content. (F) Representative Western blots showing the expression of GPX4, Bax, and Bcl-2. (G to I) Densitometric quantification of relative protein levels: (G) GPX4, (H) Bax, and (I) Bcl-2. Data are presented as means ± SD. *P < 0.05, **P < 0.01.

After the AMF + DFT combination was shown to induce a synergistic surge in intracellular ROS, the molecular pathways responsible for the observed cytotoxicity were subsequently investigated. It was hypothesized that the amplified oxidative stress could activate multiple regulated cell death pathways, particularly apoptosis and ferroptosis. Apoptotic induction was first evaluated by assessing MMP dissipation using the JC-1 fluorescent probe. A shift from red fluorescence (J-aggregates) to green fluorescence (JC-1 monomers) signifies MMP depolarization, an early hallmark of mitochondrial apoptosis. Cells subjected to AMF + DFT exhibited the most pronounced increase in green fluorescence, indicating substantial mitochondrial depolarization (Fig. 5A). Quantitative analysis corroborated this observation, showing that the green/red fluorescence ratio in the AMF + DFT group was significantly higher than in all other treatment groups (Fig. 5B).

Western blot analysis further confirmed the activation of the mitochondrial apoptotic pathway (Fig. 5F). Expression of the pro-apoptotic protein Bax was markedly up-regulated in AMF + DFT-treated cells (Fig. 5H), while expression of the antiapoptotic protein Bcl-2 was notably down-regulated (Fig. 5I). Consequently, the Bax/Bcl-2 ratio reached its highest level in the AMF + DFT group, underscoring potent induction of mitochondrial-dependent apoptosis. Collectively, these findings establish that the amplified oxidative stress generated through AMF-activated DFT nanoparticles initiates profound mitochondrial dysfunction and drives apoptosis as a primary cell death mechanism.

The activation of ferroptosis—an iron-dependent, regulated cell-death pathway characterized by excessive lipid peroxidation—was subsequently examined. Using the C11-BODIPY581/591 fluorescent probe, a pronounced increase in lipid ROS was observed in cells treated with AMF + DFT, exceeding the levels generated by DFT alone (Fig. 5C). Quantitative fluorescence analysis further confirmed this enhancement (Fig. 5D). In line with these findings, the intracellular concentration of MDA, a key terminal product of lipid peroxidation, was found to be highest in the AMF + DFT group (Fig. 5E). Moreover, Western blot analysis (Fig. 5F) revealed that GPX4—the central antioxidant enzyme protecting cells against lipid-ROS-driven ferroptosis—was markedly down-regulated after AMF + DFT treatment (Fig. 5G). These results, evidenced by pronounced lipid peroxidation and GPX4 suppression, indicate that ferroptosis was robustly activated under the combined treatment conditions.

The mechanistic investigation demonstrates that the superior efficacy of the AMF + DFT system arises from its ability to transduce an amplified ROS signal into a multipathway cytotoxic cascade. The oxidative burst simultaneously initiates apoptosis, characterized by mitochondrial depolarization and dysregulation of Bcl-2 family proteins, and ferroptosis, evidenced by extensive lipid peroxidation and GPX4 down-regulation. This dual-pathway activation accounts for the pronounced synergistic cytotoxicity observed in vitro.

### The DFT nanoplatform achieves efficient cellular uptake and in vivo tumor retention

**Fig. 6.**
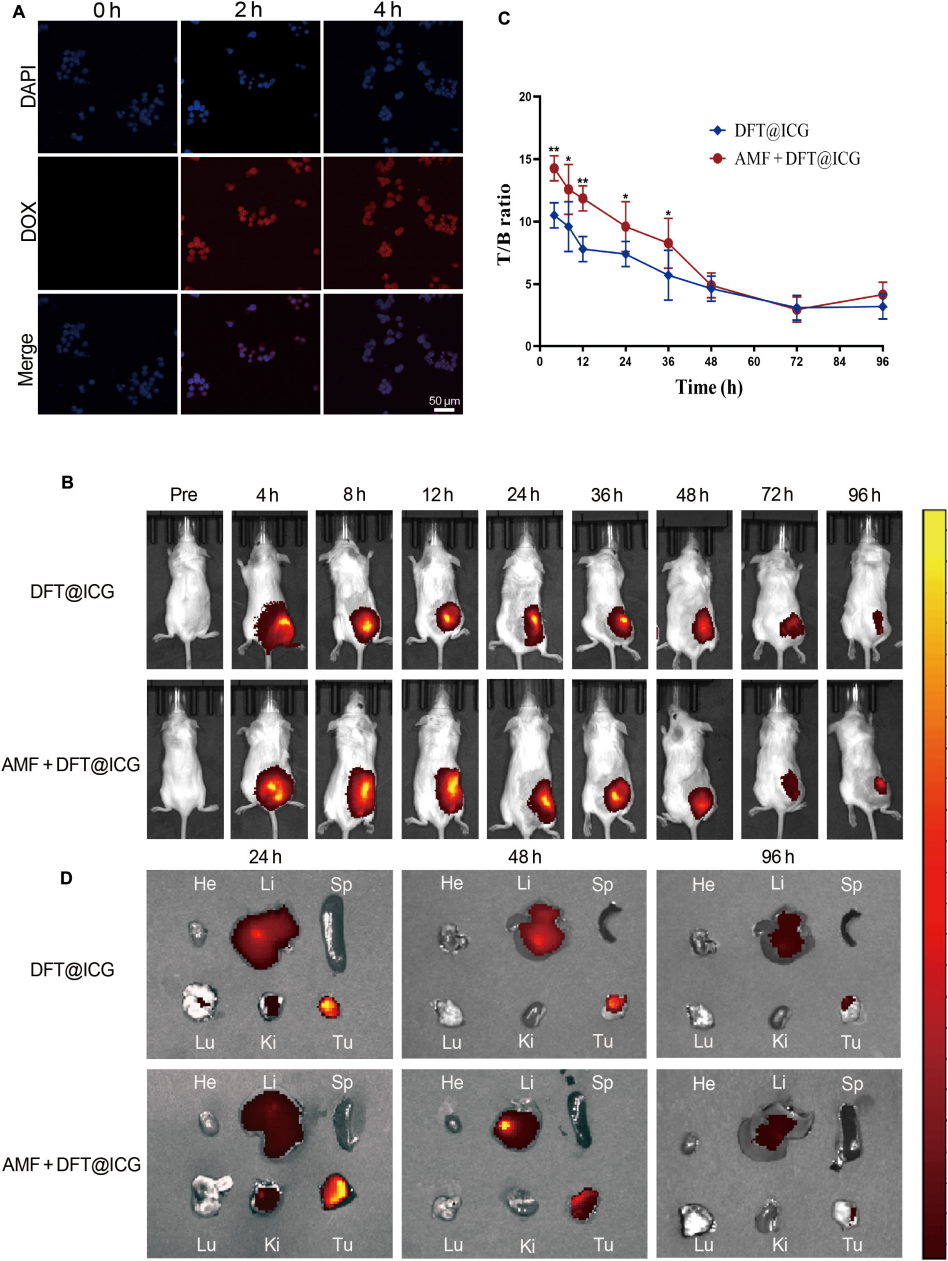
Multiscale delivery of the DFT nanoplatform from cellular uptake to in vivo tumor retention. (A) CLSM images showing time-dependent cellular uptake of DFT nanoparticles by 4T1 cells. Scale bar: 50 μm. (B) Representative in vivo whole-body near-infrared fluorescence images of DFT@ICG in 4T1 tumor-bearing mice. (C) Time-dependent T/B ratios in mice injected with DFT@ICG, comparing groups with and without localized AMF treatment. (D) Representative ex vivo fluorescence images of major organs (He, heart; Li, liver; Sp, spleen; Lu, lungs; Ki, kidneys) and tumors (Tu) harvested at indicated time points. Data are presented as means ± SD (*n* = 3). *P < 0.05, **P < 0.01.

Having elucidated the in vitro therapeutic mechanism, the successful in vivo translation of this cascade amplification paradigm critically depends on the efficient delivery and retention of the DFT nanoplatform within tumor tissues. To this end, cellular uptake was examined microscopically, followed by in vivo observation of tumor accumulation at the whole-body level. Efficient internalization by cancer cells is a prerequisite for intracellular therapeutic activity. The uptake of DFT nanoparticles by 4T1 breast cancer cells was visualized using CLSM by tracking the intrinsic fluorescence of the DOX payload. As shown in Fig. 6A, red fluorescence intensity within the cytoplasm increased progressively over 4 h, confirming time-dependent internalization and effective cellular uptake of DFT nanoparticles.

To extend these findings to in vivo conditions, we evaluated the intratumoral retention behavior of the nanoplatform using a 4T1 tumor-bearing mouse model. For near-infrared fluorescence imaging, the fluorescent dye ICG was encapsulated within DFT to form DFT@ICG. Tumor-bearing mice received intratumoral injections of DFT@ICG, and a subset of animals was exposed to a localized AMF treatment centered on the tumor site. Whole-body fluorescence imaging revealed that DFT@ICG nanoparticles remained predominantly localized within the tumor region for both groups (Fig. 6B). Quantitative T/B ratio analysis (Fig. 6C) revealed that the AMF-treated group exhibited significantly enhanced tumor retention compared with the non-AMF control. At 6 h postinjection, the T/B ratio was approximately 14 in the AMF group versus 10 in the control group (*P* < 0.01), with this statistically significant difference persisting up to 36 h (*P* < 0.05).

To further substantiate these findings, we harvested major organs and tumors at selected time points for ex vivo fluorescence imaging. The ex vivo results were consistent with the whole-body imaging observations, revealing markedly stronger fluorescence intensity in the tumors of the AMF-treated group compared with the non-AMF group (Fig. 6D). Fluorescence was also observed in the liver, attributable to clearance of nanoparticles that gradually escaped from the injection site by the mononuclear phagocyte system. Notably, tumor fluorescence remained robust and persistent in both groups, indicating effective intratumoral retention despite partial systemic clearance.

These results provide multiscale evidence from single-cell internalization to whole-animal biodistribution. The data demonstrate that the DFT nanoplatform can be efficiently internalized by cancer cells and stably retained at the tumor site. The enhanced retention conferred by localized AMF further establishes a robust foundation for subsequent in vivo therapeutic evaluation.

### The cascade amplification paradigm demonstrates potent antitumor efficacy in vivo

**Fig. 7.**
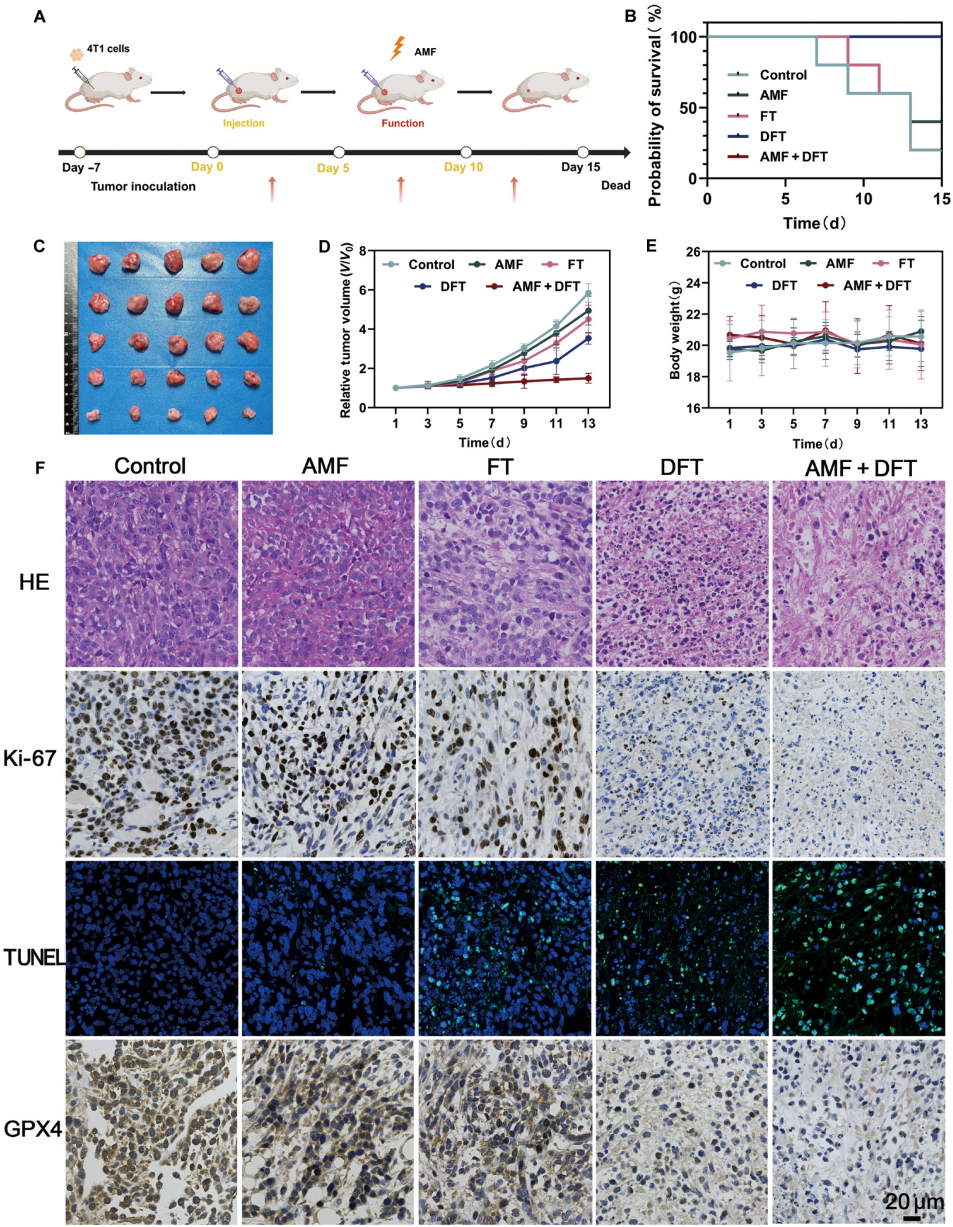
The cascade amplification paradigm demonstrates potent synergistic antitumor efficacy in vivo. (A) Schematic illustration of the in vivo experimental design. (B) Survival curves of tumor-bearing mice across treatment groups. (C) Representative photographs of excised tumors at study end point. (D) Tumor growth curves during the treatment period. (E) Body-weight changes recorded throughout therapy. (F) Representative images of HE, Ki-67, TUNEL, and GPX4 staining in tumor sections. Scale bar: 20 μm.

Having confirmed efficient in vivo tumor accumulation, the therapeutic efficacy of the DFT nanoplatform, activated through the cascade amplification paradigm, was next evaluated in a 4T1 tumor-bearing mouse model. Mice were randomized into designated treatment groups, and tumor growth was monitored over a 14-d period following the schedule outlined in Fig. 7A. The combined AMF + DFT treatment exhibited markedly superior antitumor performance compared with all control groups. Macroscopic evaluation of tumors excised at the end of the study revealed the smallest tumor masses in the AMF + DFT group (Fig. 7C), a finding quantitatively supported by tumor-volume analysis (Fig. 7D). Tumors in the untreated control group proliferated rapidly, whereas the AMF-alone and FT-alone groups displayed only marginal inhibitory effects. The DFT-alone group, representing the primed state without AMF activation, achieved a moderate tumor-growth inhibition of 40.7%. In striking contrast, the AMF + DFT group—corresponding to the full cascade amplification—achieved a potent growth inhibition of 74.6%. This nearly 2-fold improvement over DFT-alone provides compelling in vivo evidence of the therapeutic advantage conferred by AMF-mediated amplification. Moreover, survival analysis demonstrated a significant extension of median survival in the AMF + DFT group (Fig. 7B). Throughout the entire treatment course, no significant body-weight loss was observed in any group (Fig. 7E), indicating favorable systemic tolerance and excellent biocompatibility of the treatment regimen.

To verify that the observed in vivo efficacy stemmed from the same molecular mechanisms identified in vitro, we collected tumor tissues for histopathological and immunohistochemical analyses. HE staining of tumor sections from the AMF + DFT group revealed the most extensive necrosis and cellular disruption (Fig. 7F). Immunohistochemical staining for the proliferation marker Ki-67 showed a pronounced reduction in proliferating tumor cells, while TUNEL staining indicated the highest level of apoptosis within this group. Most notably, immunostaining for GPX4, a pivotal regulator of ferroptosis, demonstrated marked down-regulation in the tumors treated with AMF + DFT. This in vivo molecular signature—characterized by suppressed proliferation, enhanced apoptosis, and GPX4 down-regulation—aligns closely with the in vitro mechanistic observations, confirming that the same dual-pathway cytotoxic mechanisms operate within TME.

Collectively, the in vivo therapeutic results unequivocally demonstrate the superiority of the cascade amplification paradigm. The AMF + DFT group showed markedly enhanced therapeutic efficacy compared with the DFT-alone group. This result validates the central hypothesis that a low-intensity, nonthermal AMF can function as an external accelerator to strengthen the activity of a TME-primed nanoplatform. Histological and molecular analyses further confirmed that this amplified efficacy results from the concurrent induction of apoptosis and ferroptosis within tumor tissues. These findings indicate that the in vitro mechanistic insights are successfully translated into a complex in vivo biological environment.

### The DFT-based therapeutic platform exhibits an excellent safety profile

**Fig. 8.**
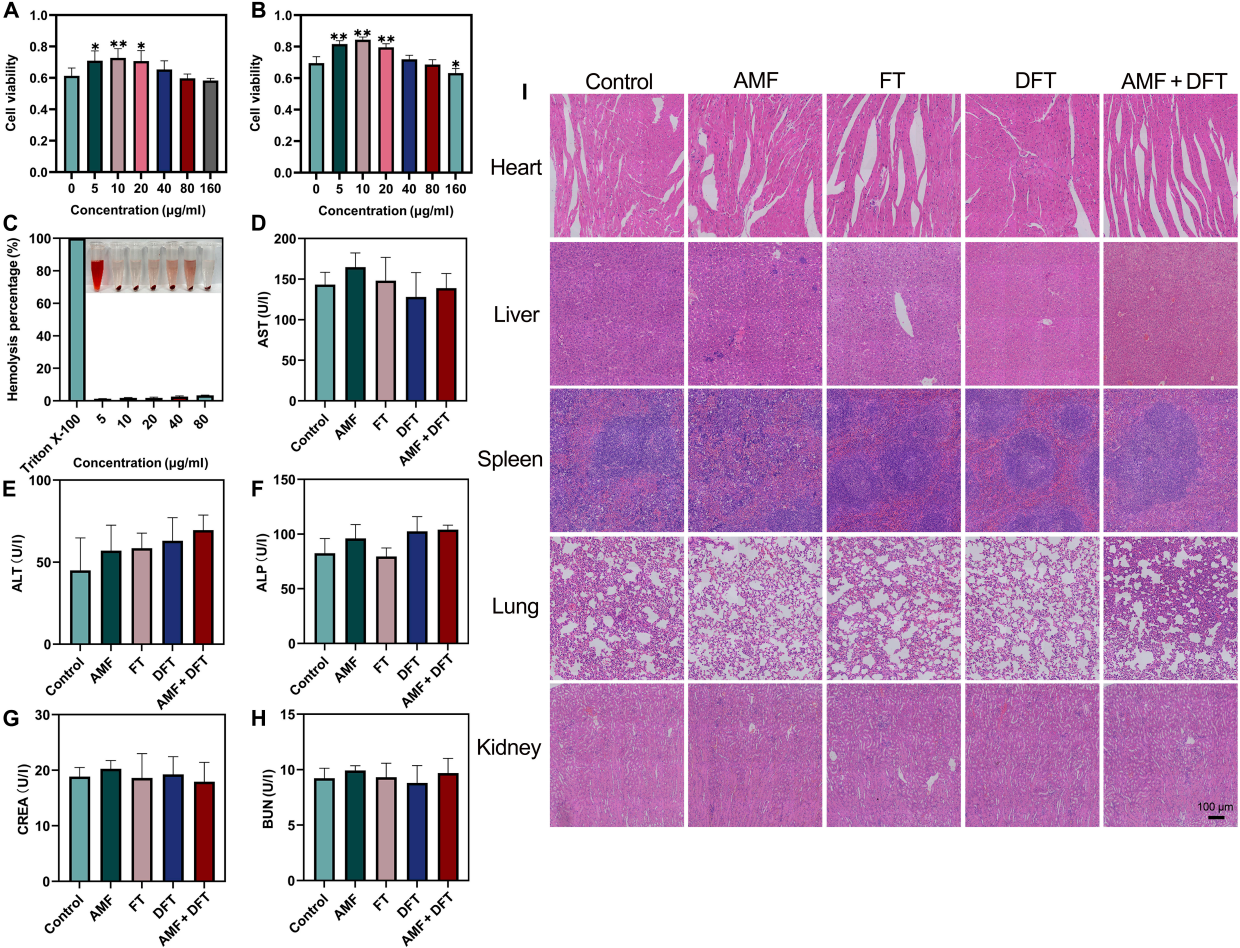
Comprehensive evaluation of biocompatibility and systemic safety. (A and B) In vitro cytocompatibility of FT and DFT nanoparticles in L929 fibroblasts. (C) In vitro hemolysis assay assessing compatibility with red blood cells. (D to H) Serum biochemical markers of liver (ALT, AST, and ALP) and kidney (BUN and CREA) function. (I) Representative HE-stained images of major organs from treated mice. Scale bar: 100 μm. Data are presented as means ± SD. *P < 0.05, **P < 0.01.

A crucial prerequisite for the clinical translation of any nanotherapeutic strategy is the establishment of a favorable safety and biocompatibility profile. Therefore, a comprehensive assessment of the DFT nanoplatform’s biocompatibility and the AMF + DFT treatment regimen was performed both in vitro and in vivo. The in vitro cytocompatibility of FT and DFT nanoparticles was first evaluated using L929 murine fibroblasts as a model of normal cells. Both formulations exhibited excellent biocompatibility, with cell viability remaining high even at concentrations exceeding those employed therapeutically in cancer cells (Fig. 8A and B). In addition, an in vitro hemolysis assay was conducted to examine the interaction of DFT nanoparticles with red blood cells. The results demonstrated minimal hemolytic activity, with a hemolysis rate below 2% at therapeutic concentrations—well within the internationally accepted safety threshold (<5%) for injectable formulations (Fig. 8C). Collectively, these findings establish that the DFT nanoparticles are intrinsically cytocompatible and hemocompatible, confirming their suitability for subsequent in vivo safety evaluation.

Building upon these in vitro findings, the systemic safety of the complete AMF + DFT treatment was evaluated in 4T1 tumor-bearing mice at the end of the efficacy study. As previously noted, no signs of overt toxicity—such as significant body-weight loss—were detected during the treatment period (Fig. 7E). To further investigate potential organ-specific toxicity, we collected blood samples for serum biochemistry analysis. Key markers of liver function (ALT, AST, and ALP) and kidney function (BUN and CREA) exhibited no significant differences between the treated groups and healthy controls, indicating that the therapy did not impair the functional integrity of these vital organs (Fig. 8D to H).

Subsequently, major organs (heart, liver, spleen, lungs, and kidneys) were excised for histopathological evaluation. HE staining revealed no evidence of tissue necrosis, inflammatory infiltration, or structural abnormalities in any treated group compared with the controls (Fig. 8I). The preservation of normal histoarchitecture, even after repeated DFT administration and localized AMF exposure, provides compelling evidence for the high systemic biosafety of the nanoplatform.

In conclusion, a comprehensive, multilevel safety assessment was conducted, encompassing in vitro cytocompatibility and hemocompatibility as well as in vivo biochemical and histopathological evaluations. The negligible hemolysis, preserved organ function, and normal splenic and pulmonary histopathology collectively indicate that macrophage uptake of DFT occurs at physiologically manageable rates without triggering blood aggregation or pathological immune overactivation. The comprehensive absence of inflammatory sequelae supports the immunological safety of the platform. The results confirm that the strong antitumor efficacy of the cascade amplification paradigm is achieved without causing significant systemic toxicity. The excellent safety profile of the AMF + DFT treatment underscores its broad therapeutic window and strongly supports its potential for future clinical translation.

## Discussion

The primary contribution of this work lies in the experimental validation of a cascade amplification therapeutic paradigm that effectively overcomes the intrinsic kinetic limitations of ROS-based nanotherapies within the heterogeneous TME. Although many advanced nanoplatforms, including multiple MOF-based architectures, have achieved sophisticated TME-responsive drug release, their therapeutic efficacy remains predominantly governed by the fluctuating biochemical conditions of individual tumors [13,26]. Consequently, such systems function in a largely passive manner, restricting therapeutic reproducibility. In contrast, the proposed cascade paradigm incorporates a crucial second layer of active, externally regulated control. In vivo investigations further revealed a striking enhancement in tumor inhibition achieved by the full cascade system (74.6%), nearly doubling that of the passive TME-primed formulation (40.7%). This quantitative evidence clearly highlights the pronounced therapeutic advantage conferred by external AMF-driven amplification. Such precise, on-demand control of therapeutic outcomes remains unattainable with conventional passive strategies.

A defining feature of this paradigm is the use of a low-intensity AMF as a nonthermal modulator of catalytic biochemistry. This mechanism fundamentally differentiates our strategy from conventional AMF-based cancer therapies that rely primarily on magnetic hyperthermia. Traditional magnetic hyperthermia typically necessitates frequencies >100 kHz or field strengths sufficient to raise tissue temperatures above 42 °C, often facing the challenge of exceeding clinically acceptable limits to attain therapeutic efficacy [19]. By contrast, the present approach circumvents this thermal constraint entirely. The selected AMF parameters (*H*·*f* ≈ 3.18 × 10^8^ A·m^−1^·s^−1^) operate at only ~66% of the conservative Brezovich clinical safety threshold (*H*·*f* < 4.85 × 10^8^ A·m^−1^·s^−1^) [27]. Remarkably, the nonthermal cascade system achieved a tumor-growth inhibition rate of 74.6%, while operating well within the established safety margin. This performance surpasses that of conventional hyperthermia-based modalities, which often approach or exceed safety boundaries to reach comparable efficacy. Indeed, some prior studies have even proposed controversial extensions of this limit to 1.9 × 10^9^ A·m^−1^·s^−1^ [28]. In situ thermometric monitoring verified that no measurable temperature elevation occurred during AMF exposure. This finding confirms that the enhanced efficacy originates from a purely nonthermal mechanism, clearly distinguishing this work from heat-dependent therapeutic strategies.

The observed 2.8-fold amplification of intracellular ROS suggests that field-induced physical effects—such as enhanced mass transport or accelerated electron transfer—are the most plausible underlying drivers of the cascade amplification. These findings demonstrate that catalytic amplification, rather than thermal ablation, represents a more potent and clinically feasible route for achieving robust antitumor efficacy without compromising safety. This nonthermal approach not only expands the therapeutic window by avoiding nonspecific heat damage but also capitalizes on the deep tissue penetration of magnetic fields, offering a distinct advantage over optical-based modalities.

Conceptually, this study establishes a therapeutic application of nonthermal magnetocatalysis, wherein a low-intensity AMF serves not as a heat source but as a remote trigger that modulates the catalytic behavior of a nanozyme in situ. Although the concept of nonthermal magnetocatalysis is still emerging [3,16], previous studies have primarily focused on fundamental or single-functional catalytic systems. In contrast, for the first time, the feasibility of integrating this subtle, field-driven modulation within a multifunctional, drug-loaded MOF platform was demonstrated, thereby enabling a coordinated therapeutic response involving the activation of dual cell-death pathways [29,30].

Our results strongly suggest that field-induced physical mechanisms—such as enhanced mass transport and accelerated electron transfer—initiate the observed catalytic amplification. However, the exact physicochemical origin of this phenomenon remains unclear and represents an exciting frontier for future investigation. Several plausible physical models merit exploration. For instance, exposure to low-intensity AMF may influence the spin states of radical intermediates within the Fenton reaction cycle, thereby reducing activation barriers or modifying oxidation pathways. Alternatively, nanoscale magneto-mechanical oscillations of the MOF framework could act as microscopic nanostirrers, enhancing the local diffusion of reactants (H₂O₂) and products (•OH) at the catalytic interface. By providing compelling in vivo biological evidence for the efficacy of nonthermal magnetocatalysis within a complex therapeutic construct, this study offers a strong impetus for continued physical investigations into these mechanisms. Understanding the interplay among magnetic field interactions, catalytic kinetics, and nanoscale energy transduction will be crucial for the rational, bottom-up design of next-generation magnetocatalytic nanoplatforms optimized for biomedical applications.

Mechanistically, the amplified oxidative burst was demonstrated to trigger a synergistic dual-pathway cell death, engaging both apoptosis and ferroptosis. The emerging strategy of combining traditional chemotherapeutics with ferroptosis inducers to overcome therapeutic resistance represents an important frontier in cancer therapy [10,31]. Most existing approaches rely on the codelivery of multiple agents or passive material degradation to induce ferroptosis. In contrast, our findings introduce a key innovation—the use of an external magnetic field as a remote, on-demand switch that selectively activates the ferroptotic pathway. Multiple lines of experimental evidence confirm the activation of ferroptosis in our system. A pronounced increase in lipid peroxidation was detected by C11-BODIPY staining and quantitative MDA assays. In parallel, a marked down-regulation of GPX4—the master regulator of the ferroptotic pathway—was also observed. By simultaneously engaging this nonapoptotic cell death process, the AMF + DFT system provides a fail-safe mechanism against apoptosis resistance, a major contributor to chemotherapy failure [32,33]. The consistent up-regulation of ferroptotic markers, in concert with the clear activation of mitochondrial apoptosis, strongly substantiates the proposed dual-pathway mechanism as the driver of the observed synergistic cytotoxicity.

Crucially, this potent synergistic cytotoxicity is highly selective toward malignant tissues. Beyond the physicochemical pH-gating effect, the safety profile is reinforced by inherent biological disparities: Normal cells, possessing robust antioxidant systems and low basal H₂O₂ levels, naturally buffer the catalytic process [34,35]. Furthermore, the biodegradation profile of the DFT nanoplatform is favorable for potential clinical translation. The MOF framework disintegrates in acidic endosomal/lysosomal compartments, releasing iron ions and porphyrin ligands. The released iron ions enter the endogenous iron metabolic pool, a process tightly regulated by ferritin and transferrin [36]. Meanwhile, the porphyrin ligands, being structurally analogous to endogenous heme, are expected to be cleared via hepatobiliary pathways. The established biocompatibility of iron-based nanomaterials further supports their safe metabolic clearance [37]. Additionally, the release kinetics are therapeutically optimized. The pH-responsive release profile—with substantial payload release occurring within 12 to 24 h under acidic conditions—is temporally synchronized with peak tumor accumulation (24 h) and the AMF application schedule. This ensures that the therapeutic payload achieves maximum bioavailability precisely when the external magnetic field trigger is applied, optimizing synergistic efficacy while minimizing systemic exposure during circulation. Such temporally coordinated release strategies represent a key design principle in modern precision nanomedicine [15].

Despite these compelling results, certain limitations delineate promising directions for future research. The cascade amplification paradigm achieved potent tumor-growth inhibition (74.6%), demonstrating strong local therapeutic efficacy. However, complete tumor eradication was not observed in all subjects during the study period. These findings indicate that future efforts should focus on translating this robust local control into a durable systemic benefit. Importantly, because ferroptosis is recognized as an immunogenic form of cell death, combining the AMF-amplified nanoplatform with immune checkpoint blockade therapy could represent a transformative strategy. Such an approach might convert the primary tumor into an in situ vaccine, enabling the development of a durable antitumor immune response that extends beyond the local treatment site.

Furthermore, several aspects merit deeper investigation. First, although our findings strongly indicate the concurrent activation of apoptosis and ferroptosis, the precise quantitative contribution of each pathway to the observed synergistic cytotoxicity was not definitively established in the present study. Future mechanistic experiments employing pathway-specific inhibitors—such as Ferrostatin-1 and Z-VAD-fmk—will be essential to disentangle their relative roles and clarify the dynamic crosstalk between these cell death modalities. Second, the in vivo evaluation was conducted over a relatively short treatment window. Therefore, extended studies are needed to assess relapse-free survival and to monitor potential long-term toxicities, including the possibility of iron overload resulting from gradual MOF degradation.

Beyond these immediate directions, the cascade amplification principle demonstrated here offers a broad conceptual framework with significant translational implications. One particularly promising frontier lies in exploring the immunomodulatory outcomes of this therapy. The induction of robust, dual-pathway cell death raises the compelling possibility of eliciting immunogenic cell death, effectively turning the tumor into an in situ vaccine [38,39]. Validating this hypothesis, along with investigating the synergy between the AMF-amplified nanoplatform and immune checkpoint blockade therapy, represents an important next step. Such studies could transform this localized intervention into a systemic and durable antitumor immune response. Ultimately, this advancement may formally establish the emerging paradigm of magneto-immuno-oncology.

More broadly, the design blueprint of coupling an endogenous biological primer with an external, noninvasive physical amplifier is not restricted to oncological applications or magnetic field modalities. This concept could be extended to other pathological contexts, such as bacterial infections or localized inflammatory diseases, and potentially integrated with alternative energy inputs including focused ultrasound. Collectively, this work lays the foundation for a new generation of remotely programmable and intelligent nanomedicines. In these systems, external physical fields are not employed for brute-force ablation but are instead harnessed as precise and tunable tools to orchestrate complex biochemical and cellular cascades.

In summary, this study successfully designed and validated an AMF-responsive iron–porphyrin MOF nanoplatform that implements a novel cascade amplification strategy for synergistic chemo-ferroptosis therapy. By integrating TME-responsive priming with remote, nonthermal AMF-triggered catalytic amplification, the system achieves potent, on-demand therapeutic efficacy with enhanced spatiotemporal control. Importantly, this nonthermal approach operates well within established clinical safety thresholds, thereby addressing the safety–efficacy considerations that limit’s conventional magnetic hyperthermia therapies. Collectively, these findings establish a powerful new paradigm in cancer nanomedicine—utilizing external physical fields not for brute-force thermal ablation but as precise, programmable modulators of catalytic biochemistry. This strategy delivers superior antitumor efficacy while maintaining a favorable safety profile. It provides a promising framework for overcoming therapeutic resistance in solid tumors. Moreover, it informs the development of the rational design of next-generation, remotely controllable, and intelligent nanotherapeutics.

## Data Availability

The data that support the findings of this study are available on request from the corresponding authors.
