# Supplementary material for: Orchestrating Chemo-Ferroptosis via Nonthermal Magnetocatalysis: A Cascade Amplification Paradigm for Breast Cancer Therapy
Source: Biomater Res. 2026 Mar 17;30:0341. doi: 10.34133/bmr.0341 (PMC12992931; doi:10.34133/bmr.0341)
Supplement: Supplementary 1 — Fig. S1 [file bmr.0341.f1.docx]

**Supporting Materials**

**Orchestrating chemo‑ferroptosis via non‑thermal magnetocatalysis: A cascade amplification paradigm for breast cancer therapy**

*Wenjie Wang*^1,2^*, Yixiao Li*^2^*, Xingyu Ma*^2^*, Tianyu Chen*^3^*, Dongyang Zhao*^2^*, Jiawei Zhai*^2^*, Xinbei Fang*^3^*, Wenting Chen^3^, Zhongling Ma*^4,5^**, Xiaojun Zhang*^2^*

^1^ Northwest University Chang An Hospital, Northwest University, Xi’an, Shaanxi, 710069, China.

^2^ School of Medicine, Northwest University, Xi’an, Shaanxi Province,710069, China;

^3^ Key Laboratory of Resource Biology and Biotechnology in Western China, Ministry of Education; Provincial Key Laboratory of Biotechnology, College of Life Sciences, Northwest University, Xi’an, Shaanxi Province,710069, China

^4^ Northwest University Chang An Hospital, Faculty of Life Sciences and Medicine, Northwest University, Xi’an, Shaanxi, 710069, China.

^5^ Department of Oncology, Chang An District Hospital, Xi'an, Shaanxi,710118, China.

*Correspondence author’s E‒mail: [zhangxj@nwu..edu.cn](mailto:zhangxj@nwu..edu.cn) (X. Zhang)

[mazhongling0524@163.com](mailto:mazhongling0524@163.com) (Z. Ma)

1. **S****upplementary methods**
   1. **Baseline cytotoxicity assessment**

The baseline cytotoxicity of the pristine FT framework and the DOX‑loaded DFT nanoplatform was evaluated in 4T1 breast cancer cells using the Cell Counting Kit‑8 (CCK‑8, New Cell Molecular Biotech, China) assay. Cells were seeded into 96‑well plates at a density of 10,000 cells per well and allowed to adhere overnight. Subsequently, the wells were treated with fresh culture medium containing various concentrations of FT or DFT (5, 10, 20, 40, or 80 μg/mL) and incubated for 24 h at 37 °C. After treatment, 10 μL of CCK‑8 solution was added to each well, followed by an additional 2 h incubation under the same conditions. Absorbance was measured at 450 nm using a microplate reader (Synergy H1, BioTek, USA). Cell viability was expressed as a percentage relative to untreated control cells.

**1.2. Systematic optimization of AMF parameters and verification of non‑thermal conditions**

Alternating magnetic field (AMF) exposure experiments were conducted using a GS‑100B signal generator (Jinan Chuangbo, China) coupled to a Helmholtz coil configuration. Culture plates were placed at the geometric center of the coils to ensure magnetic‑field homogeneity (±5%). During all experiments, the temperature at the center of each well was continuously monitored with a fiber‑optic thermometer (Neoptix Reflex, Canada) to verify non‑thermal conditions.

To determine the optimal AMF parameters for achieving maximal therapeutic synergy, three experimental factors were systematically optimized based on cell viability measured by the CCK‑8 assay:

**Magnetic field strength**: 0, 10, 15, and 20 mT (frequency: 40 kHz; duration: 40 min).

**Frequency**: 20, 30, 40, and 60 kHz (field strength:10 mT; duration:40 min).

**Exposure duration**: 0, 20, 30, 40, and 60 min (field strength:10 mT; frequency: 40 kHz).

The optimized parameters (10 mT, 40 kHz, 40 min) achieved maximal therapeutic enhancement while maintaining non‑thermal operation (ΔT < 0.2 °C throughout exposure).

**1.3. Statistical Analysis**

All quantitative data are presented as mean ± standard deviation (SD) from at least three independent experiments, unless otherwise stated. Statistical analysis was performed using one‑way ANOVA followed by Tukey’s post hoc test for multiple comparisons (GraphPad Prism, USA). A *p* < 0.05 was considered statistically significant. Statistical significance in the figures is indicated as follows: **p* < 0.05, ***p* < 0.01.

**2. Supplementary results**

**2.1. Baseline cytotoxicity profile and determination of working concentration**

Prior to investigating the synergistic effects of AMF exposure, the baseline cytotoxicity of the pristine FT framework and the DOX‑loaded DFT nanoplatform against 4T1 breast cancer cells was assessed (Fig. S1A). The pristine FT framework exhibited excellent biocompatibility, showing negligible effects on 4T1 cell viability even at concentrations up to 80 µg/mL, confirming the inherent safety of the nanocarrier. In contrast, the drug‑loaded DFT nanoplatform displayed potent, dose‑dependent cytotoxicity, with a half‑maximal inhibitory concentration (IC₅₀) of approximately 26.4 µg/mL after 24 h of incubation.

To reliably evaluate the synergistic enhancement induced by AMF, a suitable working concentration of DFT was established. A concentration of 40 µg/mL—well above the IC₅₀—was selected to provide a robust baseline therapeutic effect while maintaining sufficient margin to detect additional AMF‑induced cytotoxic amplification. This concentration was therefore employed in all subsequent AMF parameter optimization experiments.

**2.2. Systematic optimization of AMF parameters for maximal non-thermal synergy**

The primary objective of this optimization was to identify the AMF parameters that achieve an optimal balance between two competing factors: (1) maximizing DFT‑mediated synergistic cytotoxicity and (2) minimizing any confounding cytotoxicity induced by the AMF itself. To this end, the effects of magnetic field intensity, frequency, and exposure duration on the viability of 4T1 cells treated with 40 µg/mL DFT were systematically evaluated (Fig. S1B–D).

**Magnetic field intensity** (Fig. S1B):

Synergistic cytotoxicity increased as the magnetic field intensity was raised from 0 mT to 10 mT. However, further increasing the intensity to 15 mT or 20 mT produced no statistically significant improvement in DFT‑mediated cell killing, while clearly elevating the cytotoxicity of AMF alone. Therefore, 10 mT was selected as the optimal field intensity, offering maximal synergistic enhancement with minimal off‑target AMF effects.

**Frequency** (Fig. S1C):

When the frequency was varied from 20 kHz to 60 kHz, the strongest synergistic response was observed at 40 kHz. Lower frequencies yielded weaker effects, while increasing the frequency to 60 kHz conferred no additional therapeutic benefit. This finding suggests that 40 kHz may correspond to a resonant condition that maximizes non‑thermal energy coupling with the DFT nanozyme.

**Exposure duration** (Fig. S1D):

An exposure duration of 40 min resulted in the greatest DFT‑induced cytotoxicity. Notably, extending the exposure to 50 min or 60 min paradoxically increased cell viability. This phenomenon implies a potential biological “window effect,” wherein prolonged exposure to low‑intensity AMF could activate adaptive or pro‑survival mechanisms that attenuate the therapeutic efficacy. Such behavior further supports the non‑thermal, likely signaling‑mediated, nature of the AMF interaction.

Collectively, these evaluations identified the optimal AMF parameter set as 10 mT, 40 kHz, and 40 min. This combination provided the highest synergistic therapeutic benefit while minimizing intrinsic AMF cytotoxicity, ensuring that the observed antitumor effects in subsequent experiments could be confidently attributed to non‑thermal magnetocatalytic amplification. These standardized parameters were employed in all subsequent *in vitro* and *in vivo* studies.


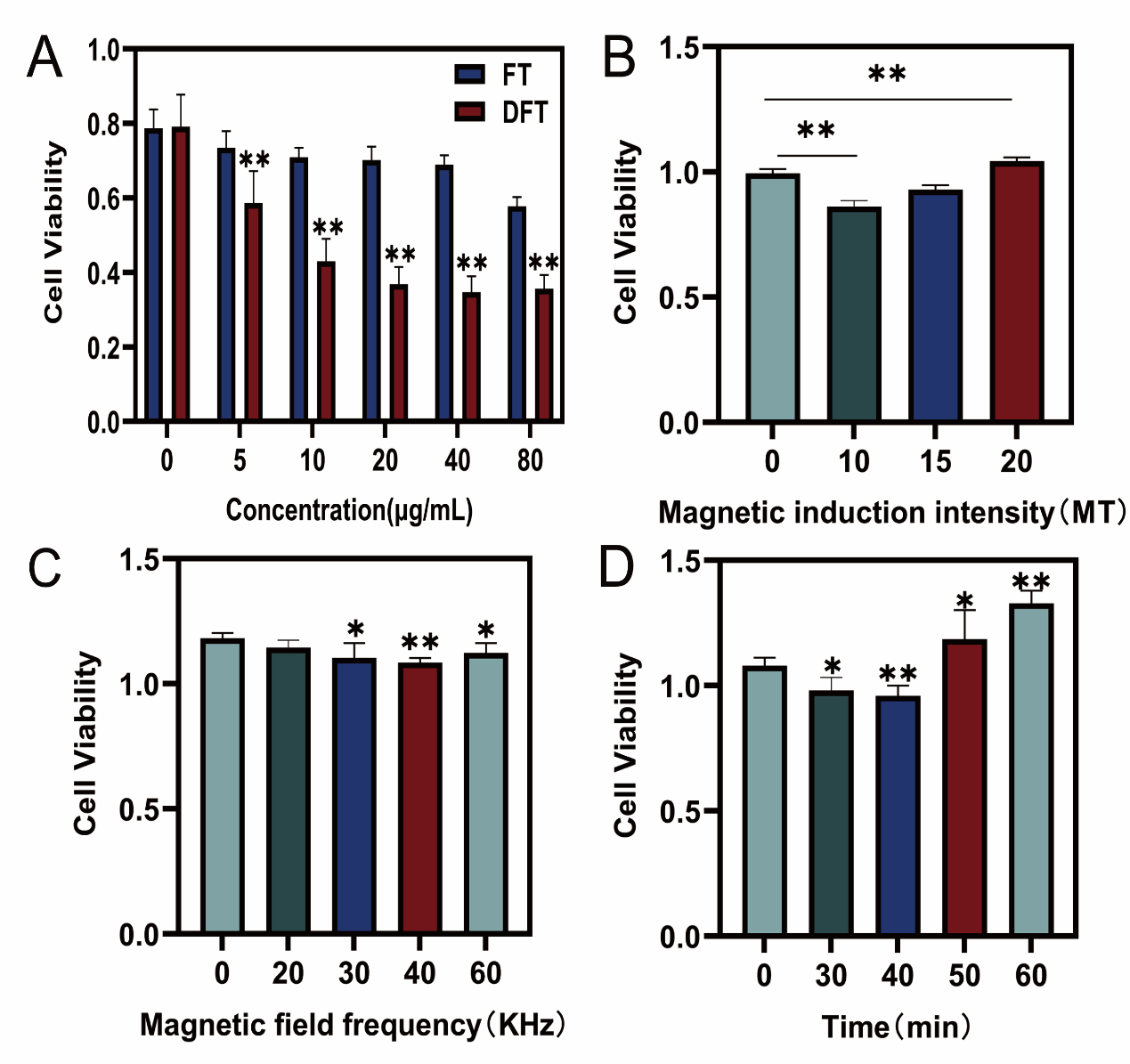


**Fig. S1. Baseline cytotoxicity and systematic optimization of AMF parameters.**

(A) Dose‑dependent cytotoxicity of the pristine FT framework and the DOX‑loaded DFT nanoplatform in 4T1 cells after 24 h of incubation. The half‑maximal inhibitory concentration (IC₅₀) of DFT was approximately 26.4 µg/mL.

(B–D) Systematic evaluation of AMF parameters to maximize synergistic cytotoxicity. The viability of 4T1 cells treated with DFT (40 µg/mL) was measured following AMF exposure with varying (B) magnetic field intensities, (C) frequencies, and (D) exposure durations. Based on these results, the optimized parameter set—10 mT, 40 kHz, and 40 min—was selected for all subsequent *in vitro* and *in vivo* experiments, as it provided maximal DFT‑mediated cytotoxicity while minimizing the inherent effects of AMF alone.
